# IGF1 promotes human myotube differentiation toward a mature metabolic and contractile phenotype

**DOI:** 10.1152/ajpcell.00654.2023

**Published:** 2024-03-04

**Authors:** Simon I. Dreher, Paul Grubba, Christine von Toerne, Alessia Moruzzi, Jennifer Maurer, Thomas Goj, Andreas L. Birkenfeld, Andreas Peter, Peter Loskill, Stefanie M. Hauck, Cora Weigert

**Affiliations:** ^1^Department for Diagnostic Laboratory Medicine, Institute for Clinical Chemistry and Pathobiochemistry, University Hospital Tübingen, Tübingen, Germany; ^2^Metabolomics and Proteomics Core Helmholtz Center Munich, German Research Center for Environmental Health, Neuherberg, Germany; ^3^NMI Natural and Medical Sciences Institute at the University of Tübingen, Reutlingen, Germany; ^4^Department for Microphysiological Systems, Institute of Biomedical Engineering, Faculty of Medicine, Eberhard Karls University Tübingen, Tübingen, Germany; ^5^German Center for Diabetes Research (DZD), Neuherberg, Germany; ^6^Institute for Diabetes Research and Metabolic Diseases of the Helmholtz Zentrum München, University of Tübingen, Tübingen, Germany; ^7^Department of Internal Medicine IV, University Hospital Tübingen, Tübingen, Germany

**Keywords:** contraction, EPS, GLUT4, human myotubes, proteomics

## Abstract

Skeletal muscle mediates the beneficial effects of exercise, thereby improving insulin sensitivity and reducing the risk for type 2 diabetes. Current human skeletal muscle models in vitro are incapable of fully recapitulating its physiological functions especially muscle contractility. By supplementation of insulin-like growth factor 1 (IGF1), a growth factor secreted by myofibers in vivo, we aimed to overcome these limitations. We monitored the differentiation process starting from primary human CD56-positive myoblasts in the presence/absence of IGF1 in serum-free medium in daily collected samples for 10 days. IGF1-supported differentiation formed thicker multinucleated myotubes showing physiological contraction upon electrical pulse stimulation (EPS) following *day 6*. Myotubes without IGF1 were almost incapable of contraction. IGF1 treatment shifted the proteome toward skeletal muscle-specific proteins that contribute to myofibril and sarcomere assembly, striated muscle contraction, and ATP production. Elevated PPARGC1A, MYH7, and reduced MYH1/2 suggest a more oxidative phenotype further demonstrated by higher abundance of proteins of the respiratory chain and elevated mitochondrial respiration. IGF1-treatment also upregulated glucose transporter (GLUT)4 and increased insulin-dependent glucose uptake compared with myotubes differentiated without IGF1. To conclude, addition of IGF1 to serum-free medium significantly improves the differentiation of human myotubes that showed enhanced myofibril formation, response to electrical pulse stimulation, oxidative respiratory capacity, and glucose metabolism overcoming limitations of previous standards. This novel protocol enables investigation of muscular exercise on a molecular level.

**NEW & NOTEWORTHY** Human skeletal muscle models are highly valuable to study how exercise prevents type 2 diabetes without invasive biopsies. Current models did not fully recapitulate the function of skeletal muscle especially during exercise. By supplementing insulin-like growth factor 1 (IGF1), the authors developed a functional human skeletal muscle model characterized by inducible contractility and increased oxidative and insulin-sensitive metabolism. The novel protocol overcomes the limitations of previous standards and enables investigation of exercise on a molecular level.

## Graphical abstract

**Figure:**
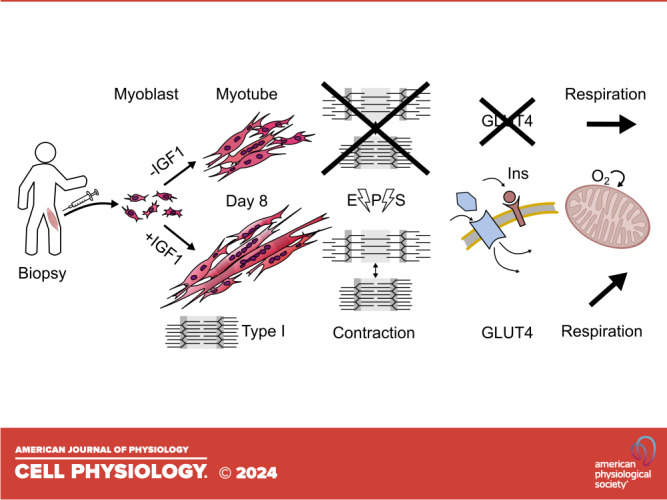

## INTRODUCTION

Increased physical activity is a major part of most lifestyle intervention programs as it can preserve insulin sensitivity in healthy persons and restore compromised insulin sensitivity in subjects with prediabetes and diabetes (1–3). As the predominantly utilized organ, skeletal muscle and its relevance for the beneficial effects of physical activity have been extensively characterized (4–6). Adaptations involved in this metabolic benefit are increased skeletal muscle mass, capillarization, mitochondrial content and respiration thereby supporting skeletal muscle glucose disposal in response to insulin (4, 7, 8). Skeletal muscle also represents the organ responsible for over 80% of insulin-dependent glucose uptake in the human body (5, 9). In addition, it contributes to systemic adaptive processes by releasing myokines (10) and myometabokines to regulate signaling pathways involved in exercise adaptation in an auto-, para-, or endocrine manner (11). However, our understanding of the communication between the exercising muscle and different organs and cell types is still incomplete. This is particularly true for the systemic, health-promoting effects of exercise (12). To study biological processes such as exercise on a whole body level and its multiorgan response and interconnection on a molecular level requires physiologically relevant models. To answer many research questions, screening the impact of compounds or metabolites on a system and sampling of tissue biopsies of internal organs cannot be performed in humans directly that leaves researchers with two options. Common mouse models, while allowing to study the interplay between organ systems come with inherent ethical limitations and, in some cases, limited transferability of findings to the human system due to metabolic, molecular, and anatomical differences (13, 14). Alternatively, and close to the system of interest, in vitro models of primary human cells are used and their interplay in cocultures or organ-on-chip models have gained interest in recent years especially in light of the first of the 3Rs (Replacement, Reduction, and Refinement) to replace animal models (15, 16).

In vitro protocols for differentiation of myotubes from primary human satellite cells have existed for decades (17–19) and have been further optimized over the years from using rat or human brain extract (20, 21) over 2% serum (horse or fetal calf) (22, 23) to serum-free protocols (24, 25). They consistently yield long, fused, multinucleated myotubes that recapitulate some aspects of myofibers in vivo and are a widely used tool to answer specific molecular questions. Nevertheless, the current in vitro differentiation models are yet limited with relation to the physiological functions of myofibers in vivo especially regarding muscle contraction and key features of its metabolic function, e.g., mitochondrial respiration and glycemic control (26). Human myotubes barely contract during electrical pulse stimulation (EPS) (27, 28), they predominantly produce ATP by glycolysis, rather than mitochondrial oxidative phosphorylation (29), the insulin-responsive glucose uptake is reduced, and the ratio of glucose transporter (GLUT)4 to GLUT1 expression is low compared with adult skeletal muscle (30, 31). During stem cell and precursor cell differentiation, growth factors are needed to guide proper differentiation. For promotion of contractility, the growth factor insulin-like growth factor 1 (IGF1) is a good candidate. IGF1 is secreted by myofibers during muscle movement in vivo and is responsible for hypertrophy, and production of muscle-specific and contractile apparatus proteins (32–34). In addition, IGF1 already could have played a small part in previous promising yet complicated multistep differentiation attempts to overcome some of the functional limitations of primary human myotubes in vitro (25).

The aim of the study was to utilize IGF1 to develop a more physiological myotube model from primary human precursor cells in vitro. We used a serum-free differentiation protocol that was superior to medium containing 2% serum to differentiate myotubes from primary human myoblasts supplemented with or without 100 ng/mL of IGF1 corresponding to total IGF1 concentrations in human plasma (24, 35, 36). Over a time course of 10 days, we took daily samples to characterize differences between the two differentiation protocols in the proteome and on transcript level, used immunohistochemistry to visualize cell fusion and structural proteins, applied immunoblotting and glucose uptake assay to study the insulin responsiveness, measured mitochondrial respiration using Seahorse, and assessed the capability to functionally contract by EPS and video analysis.

## MATERIALS AND METHODS

### Cell Culture

**Figure 1.**
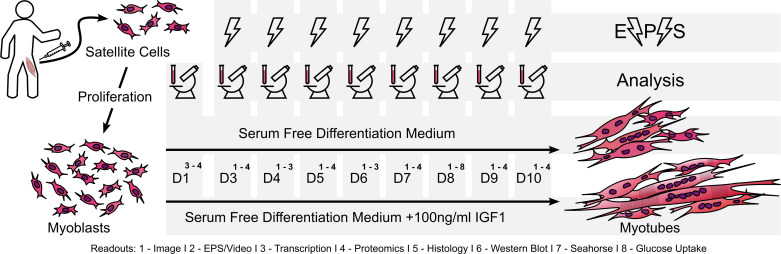
Experimental design. Primary human satellite cells isolated from vastus lateralis muscle biopsies were proliferated to myoblasts and differentiated into myotubes for 10 days in serum-free differentiation medium in the presence or absence of 100 ng/mL insulin-like growth factor 1 (IGF1). On *day 1* and daily from *day 3* myotubes were sampled and analyzed. Cells were imaged, electrical-pulse-stimulation (EPS) was performed, and transcription was analyzed daily from *day 3* to *day 10*. From samples obtained on *day 1* proteomic analysis were performed and transcription was analyzed. From samples obtained on *days 3*, *5,* and *7–10* proteomic analyses were performed. Functional analysis including immunostaining, phospho-Western-blotting, respiratory measurement by Seahorse and glucose uptake was conducted on *day 8*.

Primary human myoblasts were obtained from muscle biopsies as described previously (37). In brief, muscle biopsies were taken from the lateral portion of the vastus lateralis of the quadriceps femoris after local anesthesia (2% Scandicaine; AstraZeneca, Germany) under sterile conditions using a Bergström needle (Pelomi Medical, Denmark) with suction. Biopsy donors were participants of two previous exercise intervention studies (38, 39) and only baseline biopsies were used. For the time course experiment (Fig. 1), biopsies from a total of *n* = 4 donors [2 females, 2 males, age 36 ± 12 (25–52), body mass index (BMI) 34 ± 5 (27.62–39.98)] were used and in total *n* = 14 donors [10 females, 4 males, age 33 ± 9 (21–52), BMI 30 ± 4 (23.61–39.98)] for the additional analysis of mitochondrial respiration, insulin signaling, glucose uptake, and immunofluorescence staining. All participants gave written informed consent and the study protocols were approved by the ethics committee of the University of Tübingen and in accordance with the declaration of Helsinki. Myoblast isolation and enrichment of CD56+ myoblasts by magnetic bead cell sorting are described in the study by Hoffmann et al. (24). In brief, human satellite cells were released by collagenase digestion and seeded on 15-cm dishes coated with GelTrex (Thermo Fisher Scientific, Germany). After two rounds of proliferation in cloning medium [α-MEM:Ham’s F-12 (1:1), 20% (vol/vol) FBS, 1% (vol/vol) chicken extract, 2 mM l-glutamine, 100 U/mL penicillin, 100 µg/mL streptomycin, 0.5 µg/mL amphotericin B], CD56-positive myoblasts were enriched (>90%) using MACS microbeads and LS columns (Miltenyi Biotech, Germany), according to the manufacturer’s protocol, with a 30-min incubation. They were then stored in the gaseous phase of liquid nitrogen. Cell culture surfaces were prepared with a nongelling thin-layer GelTrex coating. Myoblasts (*passage 3* after isolation, *passage 1* after enrichment) were proliferated in cloning media until 90% confluency. Myotube differentiation was induced on *day 0* and maintained for 10 days in fusion media [α-MEM, 2 mM l-glutamine, 50 µM palmitate, 50 µM oleate (complexed to BSA with a final BSA concentration of 1.6 mg/mL in medium), 100 µM carnitine] with or without supplementation of 100 ng/mL (13.16 nmol/L) IGF1 (human recombinant IGF1, I3769, Sigma-Aldrich, Germany). Medium was changed three times per week and 48 h before harvest. For the time course experiment, cells were harvested on *day 1* of myotube differentiation and every day from *day 3* to *day 10* (Fig. 1). Mycoplasma-free culture conditions and cells are regularly controlled for using the MycoAlert Mycoplasma Detection Kit (Lonza, Switzerland).

### Electrical Pulse Stimulation

For electrical pulse stimulation (EPS), cells were cultured in compatible six-well plates (Falcon, Corning) in parallel. From *day 3* of myotube differentiation, EPS was performed for 4 h at 1 Hz, 2 ms, 25 V using C-Pace EP (Ionoptix) and three videos of 15 s at 60 fps/group and donor were taken in random spots of each well using an Axiovert 40C (Carl Zeiss Microscopy, Germany) with Flexacam C3 Camera (Leica, Germany). To achieve tetanic contraction, EPS was performed at 10 Hz, 2 ms, 25 V for 5 s followed by a resting phase.

### Video Analysis and Movement Index

Myotube contraction speed, directionality, and adherence to EPS frequency were analyzed using the open heart ware (OHW) software (40). A total of 189 randomly taken videos, three videos per group and timepoint over the time course from *day 4* to *day 10* were evaluated by six independent blinded observers that evaluated amount of movement based on reference videos with a value between 0 and 3. Value 0 corresponds to no movement, 1: slight contraction/movement of a partial structure/myotube, 2: clear contraction/movement of a partial structure/myotube or slight contraction/movement of a complete structure/myotube, 3: clear contraction/movement of a complete structure/myotube. For each timepoint, condition, and donor, a single movement index between 0 and 9 was calculated by first calculating the mean value per video over all observers and subsequently calculating the sum of all three videos per timepoint, condition, and donor. Movement index of all *n* = 4 donors were used for statistical evaluation.

### Proteomics

#### Mass spectrometry sample preparation.

Cells were lysed in RIPA buffer (25 mM Tris pH 7.6, 150 mM NaCl, 3.5 mM SDS, 12.1 mM sodium deoxycholate, 1% vol/vol Triton X100) and incubated at 4°C for 30 min. Lysates were then subjected to ice-cold sonication bath for 30 s and another incubation (4°C, 15 min). The protein concentration of the individual samples was determined using bicinchoninic acid (BCA) assay following the instruction manual (Pierce Biotechnology). BSA was used as an internal standard. Ten micrograms of sample were enzymatically proteolyzed using a modified filter-aided sample preparation (FASP) protocol as described by Grosche et al. (41) and Wiśniewski et al. (42). Peptides were stored at −20°C until mass spectrometry (MS) measurement.

#### MS measurements.

LC-MSMS analysis was performed in data-dependent acquisition (DDA) mode. MS data were acquired on a Q-Exactive HF-X mass spectrometer (Thermo Fisher Scientific, Germany) coupled online to a nano-RSLC (Ultimate 3000 RSLC; Dionex). Tryptic peptides were automatically loaded on a C18 trap column [300 µm inner diameter (ID) × 5 mm, Acclaim PepMap100 C18, 5 µm, 100 Å, LC Packings] at 30 µL/min flow rate. For chromatography, a C18 reversed phase analytical column (nanoEase MZ HSS T3 Column, 100 Å, 1.8 µm, 75 µm × 250 mm, Waters) at 250 nL/min flow rate in a 95-min nonlinear acetonitrile gradient from 3 to 40% in 0.1% formic acid was used. The high-resolution (60,000 full width at half-maximum) MS spectrum was acquired with a mass range from 300 to 1,500 mass-to-charge ratio (*m/z*) with automatic gain control target set to 3 × 10^6^ and a maximum of 30-ms injection time. From the MS prescan, the 15 most abundant peptide ions were selected for fragmentation (MSMS) if at least doubly charged, with a dynamic exclusion of 30 s. MSMS spectra were recorded at 15,000 resolution with automatic gain control target set to 5 × 10^2^ and a maximum of 50-ms injection time. The normalized collision energy was 28, and the spectra were recorded in profile mode.

#### MS data processing and protein identification.

Proteome Discoverer software (Thermo Fisher Scientific; v.2.5) was used for peptide and protein identification via a database search (Sequest HT search engine) against Swissprot human database (Release 2020_02, 20349 sequences), considering full tryptic specificity, allowing for up to one missed tryptic cleavage site, precursor mass tolerance set to 10 ppm, fragment mass tolerance to 0.02 Da. Carbamidomethylation of cysteine was set as a static modification. Dynamic modifications included deamidation of asparagine and glutamine, oxidation of methionine, and a combination of methionine loss with acetylation on protein N-terminus. Percolator was used for validating peptide spectrum matches and peptides, accepting only the top-scoring hit for each spectrum, and satisfying the cut-off values for false discovery rate (FDR) < 1%, and posterior error probability <0.05. The final list of proteins complied with the strict parsimony principle. Protein identification with a Sequest HT score of below 1.6 were excluded from further analysis.

#### MS label-free quantification.

The quantification of proteins was based on the area under the curve of the abundance values for unique peptides. Abundance values were normalized against total abundance to account for sample loading errors. The protein abundances were calculated averaging the abundance values for up to 50 admissible peptides. The final protein ratio was calculated as median of all peptide comparisons between all samples. The statistical significance of the ratio change was ascertained using the *T* test approach described (43), which is based on the presumption that expression changes are screened for proteins that are just a few compared with the number of total proteins being quantified. The quantification variability of the nonchanging “background” proteins can be used to infer which proteins change their expression in a statistically significant manner.

To ensure robustness of results, a change in protein abundance was considered significant with a Benjamini–Hochberg (BH) corrected *P* value below 0.05 only if detected in at least three of four donors in at least one experimental group.

### Gene Expression

RNA was extracted using the RNeasy kit (Qiagen, Germany). For mRNA detection, reverse transcription was carried out with the Transcriptor first strand synthesis kit (Roche, Switzerland). Expression was measured using QuantiFast SYBR Green PCR Mix and QuantiTect Primer Assays [*MYH1*: QT01671005; *MYH2*: QT00082495; *MYH7*: QT00000602; *PPARGC1A* (*PGC1α*): QT00095578; *GLUT1* (*SLC2A1*): QT00068957; *GLUT4* (*SLC2A4*): QT00097902; *RPS28*: QT02310203; *TBP*: QT00000721; Qiagen, Germany] in a LightCycler 480 (Roche, Switzerland). mRNA standards for PCR were generated by purifying PCR-product (MinE-lute PCR Purification Kit, Qiagen, Germany) and 10-fold serial dilution.

### Immunofluorescence Staining

Myotubes cultured on one-well cell culture chamber on PCA slide (94.6140.102 Sarstedt, Germany) were fixed on *day 8* with ROTI-Histofix 4% (Roth, Germany) for 20 min at room temperature before storage at 4°C. Fixed myotubes were blocked and permeabilized in 3% (m/m)-BSA solution with 0.1% (m/m) Digitonin (D5628, Sigma-Aldrich, Germany) in Dulbecco’s phosphate-buffered saline (DPBS) (Thermo Fisher Scientific, Germany) for 30 min at room temperature. Proteins were stained with monoclonal anti-Myosin, MHC-fast, Clone MY-32 (1:2,000, M4276, Sigma-Aldrich, Germany, RRID:AB_477190) and monoclonal anti-Myosin, MHC-slow, Clone NOQ7.5.4D (1:4,000, M8421, Sigma-Aldrich, Germany, RRID:AB_477248) primary antibodies at 4°C overnight, Goat anti-Mouse IgG1 Cross-Adsorbed Secondary Antibody, Alexa Fluo 488 (1:2,000, A21121, Invitrogen, RRID:AB_2535764) secondary antibody, DAPI (1:500, Invitrogen) and Rhodamine Phalloidin (1:300, Invitrogen) for 2 h at room temperature and imaged using the ApoTome System (Carl Zeiss Microscopy, Germany).

### Myotube Width and Fusion Index

Width measurements were performed in randomly taken images of myotubes differentiated for 8 days, in the absence or presence of IGF and stained with MYH1 and MYH2 antibody (green) and with DAPI (blue). Width was measured by calculating the mean of three width measurements perpendicular to the longitudinal direction of the fused myotube within a predefined length of 800 µm to ensure comparability between groups and images. Width measurements were performed using ImageJ Version: 2.14.0/1.54f in a total of *n* = 96 randomly taken images, 48/group from *n* = 3 individual donors. The fusion index was calculated by dividing the number of nuclei in MYH1/2-positive myotubes by the total number of nuclei and myotubes within a randomly predefined area of 800 µm × 600 µm. Fusion index was calculated for a total of *n* = 30 randomly taken images, in 15 images/group from *n* = 3 individual donors.

### Mitochondrial Respiration

Myoblasts were cultured directly on the Seahorse assay plates (Seahorse XFe24 FluxPak, Agilent) with 20,000 cells/well. On *day 8* of myotube differentiation, medium was removed and cells were washed according to the manufacturer’s protocol with Seahorse assay medium consisting of DMEM (103575-100, Agilent) with 10 mM glucose, 2 mM l-glutamine, and 1 mM pyruvate. After calibration of the Seahorse XFe24 Analyzer (Agilent), respiration was measured using freshly prepared substrates in Seahorse assay medium: 10 µM oligomycin (*port 1*), 20 mM FCCP (*port 2*), and 5 µM rotenone and 5 µM antimycin A (*port 3*). After measurement, cells were lysed in RIPA buffer [25 mM Tris pH7.6, 150 mM NaCl, 3.5 mM SDS, 12.1 mM sodium deoxycholate, 1% (vol/vol) Triton X100] and total protein content determined in 10 µL lysate by BCA assay (Pierce Biotechnology) in duplicates. Oxygen consumption rate (OCR) and extracellular acidification rate (ECAR) were normalized to protein content. Seahorse measurement was performed in quadruplicates of *n* = 4 individual donors. The calculation of mitochondrial respiration was carried out according to manufacturer’s protocol using Wave software. Total oxygen consumption (OCR) is the area under the curve (AUC) for the total measurement. Basal respiration is baseline OCR (no injection) minus nonmitochondrial respiration (OCR after Rotenone and Antimycin A injection). Proton leak is OCR after Oligomycin injection minus nonmitochondrial respiration. ATP production is the difference between basal respiration and proton leak. Uncoupled (maximal) respiration is OCR after FCCP injection minus nonmitochondrial respiration. Spare capacity is the difference between uncoupled respiration and basal respiration. The energy map was drawn for each measurement by plotting OCR/ECAR and means ± SD after calculation of the quotient.

### Immunoblotting

Protein lysates were prepared in RIPA buffer [25 mM Tris pH 7.6, 150 mM NaCl, 3.5 mM SDS, 12.1 mM sodium deoxycholate, 1% (vol/vol) Triton X100] containing cOmplete EDTA-free protease inhibitor (Roche Diagnostics, Switzerland) and phosphatase inhibitor (1 mM NaF, 0.5 mM sodium pyrophosphate, 1 mM β-glycerophosphate, 1 mM sodium orthovanadate). Protein concentration was determined by BCA assay (Pierce Biotechnology) and samples heated in Laemmli sample buffer for 5 min to 95°C. Sodium dodecyl sulfate polyacrylamide (7.5–15%) gradient gel electrophoresis and semidry electroblotting [transfer buffer: 48 mM Tris, 39 mM glycine, 1.3 mM SDS, and 20% (vol/vol) methanol] were performed using nitrocellulose membranes (Whatman, UK). NET buffer [150 mM NaCl, 50 mM Tris/HCl, pH 7.4, 5 mM EDTA, 0.05% (vol/vol) Triton X-100, and 0.25% (m/m) gelatin] was used for blocking. Proteins were detected using GLUT4 (1:2,000, PA5-23052, Invitrogen, RRID:AB_11153908), AKT (1:500, 610860, BD, RRID:AB_398179), pAKT(Ser-473) (1:1,000, 9271 L, cell signaling, RRID:AB_329825), myosin heavy chain fast (MyHfast), Clone MY-32 (1:2,000, M4276, Sigma-Aldrich, Germany, RRID:AB_477190), and GAPDH (1:20,000, ab8245, abcam, RRID:AB_2107448) primary antibodies, overnight at 4°C, and IRDye 680RD Goat anti-Mouse IgG secondary antibody (1:20,000, 926-68070, Li-COR, RRID:AB_10956588) and IRDye 800CW Goat anti-Rabbit IgG secondary antibody (1:10,000, 926-32211, Li-COR, RRID:AB_621843) for 2 h at room temperature. Signals were detected on an Odyssey scanner (Li-COR), quantification of signal intensity was performed with ImageStudio. GLUT4 protein levels were normalized on GAPDH, pAKT referred to total AKT.

### Glucose Uptake

Glucose uptake was determined using the Glucose Uptake-Glo assay (Promega) following the protocol provided by the manufacturer. Myotubes grown on 24-well plates were fasted for 3 h in glucose-free DMEM (103575-100, Agilent). Incubation with 100 nM insulin (Roche Diagnostics, Switzerland) was performed for 1 h at 37°C followed by 1-h incubation at room temperature after adding 10 mM 2-deoxyglucose. Luminescence was measured using the GloMax Multi Detection System (Promega) after 1-h incubation at room temperature with the provided detection reagent. Obtained relative light units (RLU) were donor normalized.

### Data Analysis and Statistics

Linear regression models were analyzed with R4.1.1/RStudio (44). Normality was tested by Shapiro–Wilk test from the R package “stats” (v.3.6.3) and non-normal data were log-transformed. For correction of multiple testing, *P* values were adjusted with Benjamini–Hochberg FDR < 5% (BH). Plotted protein abundances based on proteomic analysis reflect median values over all detected peptides. Data were given as means ± SD and BH corrected *P* values <0.05 were considered significant. Differences between all timepoints were assessed using one-way ANOVA with Fisher’s least-significant difference (LSD) post hoc test or Bonferroni correction for multiple comparisons when appropriate. Graphs were made using the R packages “ggplot2” (v.3.3.2) and “ggrepel” (v.0.9.1) and figures assembled using InkScape (v1.0). Functional enrichment analysis was carried out online using https://biit.cs.ut.ee/gprofiler/with *P* < 0.05 as threshold and g:SCS as method for multiple testing correction. Homo sapiens was chosen as organism and analysis was performed using the databases from gene ontology biological process (GO:BP), cellular components (GO:CC), molecular functions (GO:MF), Reactome (REAC), Kyoto Encyclopedia of Genes and Genomes (KEGG), Wikipathways (WP), and human protein atlas (HPA). Ingenuity pathway analysis (IPA) for upstream regulators was performed based on proteomics datasets comparing +IGF versus −IGF.

## RESULTS

### Improved Differentiation and Contraction in +IGF Myotubes

**Figure 2.**
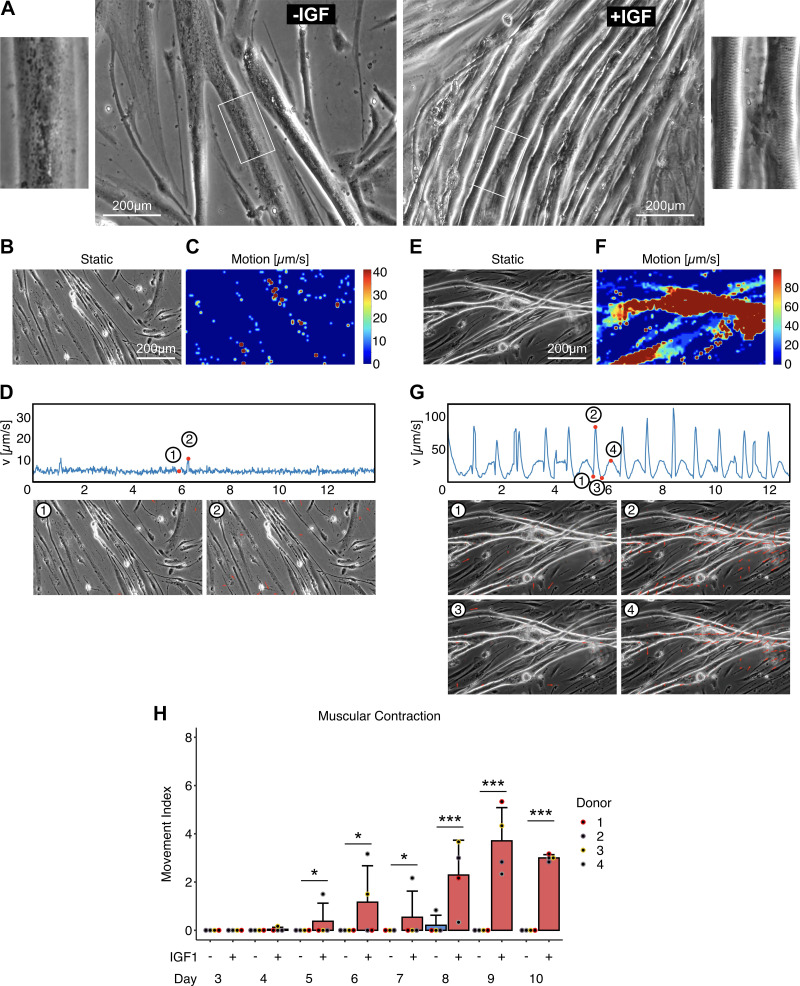
Human myotube morphology and contraction. Primary human myotubes differentiated in the presence or absence of insulin-like growth factor 1 (IGF1) to analyze morphology and capability for contraction in response to electrical-pulse-stimulation (EPS). *A*: microscopic images of differentiated myotubes from a representative donor on *day 8* of differentiation. *B*–*G*: representative output of the video analysis with the open heart wave (OHW) program for myotubes differentiated without (*B*–*D*) or with IGF1 (*E*–*G*). Static frame of the video (*B* and *E*); heatmap representing degree and area of movement (*C* and *F*); movement over time curve with static frames and arrow plots visualizing contraction in response to EPS at 1 Hz (*D* and *G*). *H*: contractability by EPS was quantified by calculating the movement index (0–9) based on randomly taken videos between *day 3* and *day 10* in myotubes differentiated without (blue bars) or with IGF1 (red bars). Bars represent means ± SD, individual data points are depicted. Scale bars represent 200 µm. Significant differences were assessed using one-way ANOVA with Fisher’s least-significant differece (LSD) post hoc test, *P < 0.05, ***P < 0.001, *n* = 4 individual donors.

Microscopic evaluation of differentiating myotubes revealed optical differences between myotubes differentiated with (+IGF) or without IGF1 (−IGF) starting around *days 4* and *5* (Supplemental Fig. S1). Although both protocols yielded elongated fused myotubes, from *days 5* to *6* thicker myotubes with bigger diameters were visible in the presence of IGF1. From *days 6* to *8* these myotubes showed a more pronounced three-dimensional phenotype, significantly greater width and a slightly elevated fusion index compared with the myotubes fused without IGF1 (Supplemental Fig. S1, *A* and *B*). On *day 8*, a clear striated pattern representing the contractile apparatus was only visible in the presence of IGF1 as depicted for a representative donor (Fig. 2*A*). When we applied electrical pulse stimulation (EPS) to myotubes differentiated without IGF1, typically no contraction was visible (Fig. 2, *B–D*). The heatmap of moving areas generated by the OHW software shows only areas outside of cells that represent background and air bubbles moving (Fig. 2*C*), which is also represented by the basically flat line representing movement speed over time (Fig. 2*D*). In contrast, the myotubes differentiated with IGF1 showed pronounced contraction (Fig. 2, *E* and *F*) that nicely adhered to the 1 Hz provided by EPS (Fig. 2*G*). The movement curve (Fig. 2*G*) demonstrates controlled and fast contraction from relaxed resting position 1 to fully contracted at 3 with the most movement at 2 toward full contraction at 3 and a slower relaxation after the end of the pulse from 3 over the most movement toward relaxation at 4 to the next relaxed resting position (Fig. 2*G*). We quantified myotube contraction by calculating the movement index, screening 189 videos, three videos per group and timepoint randomly taken (Fig. 2*H*). Contraction started to be visible around *day 5* of differentiation with IGF1 increasing in frequency and intensity until *days 8* and *9* where physiological contraction is visible in all donors differentiated with IGF1 whereas cellular movement in response to EPS was almost completely absent in myotubes differentiated without IGF1 (Fig. 2*H*). For reference, we provide videos representing best, average, and worst contractions found in myotubes differentiated with IGF1 (Supplemental Videos S1–S7) as well as representative videos showing myotubes differentiated without IGF1 during EPS (Supplemental Videos S8–S10). We frequently observed spontaneous contractions only in myotubes differentiated with IGF1 (Supplemental Videos S11–S13). In one case we managed to film spontaneous contraction followed by controlled contraction with EPS (Supplemental Video S13). By increasing stimulation to 10 Hz, myotubes differentiated with IGF1 can perform tetanic contractions repeatedly, demonstrating the potential of our model to functionally simulate different exercise modalities in vitro (Supplemental Video S14). In summary, differentiation of primary human myoblasts with IGF1 supplementation leads to visibly thicker myotubes with striated pattern formation and functional contractibility induced by EPS.

### Myotubes with Altered Proteome

**Figure 3.**
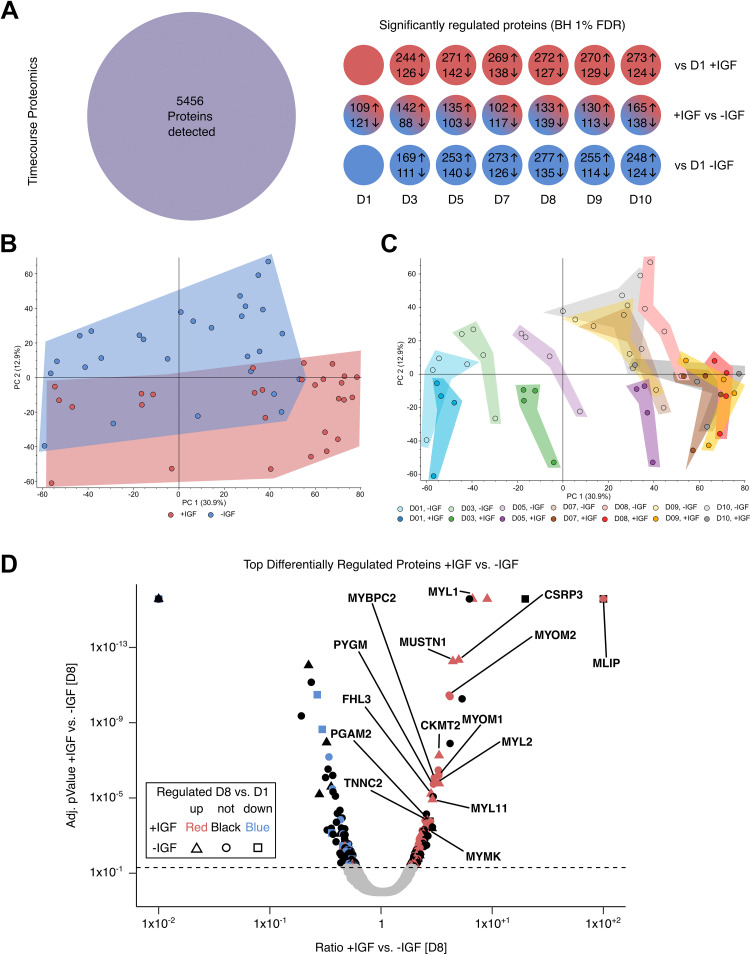
Proteomic analysis of human myotube differentiation. Primary human myotubes differentiated in the presence or absence of insulin-like growth factor 1 (IGF1) were subjected to proteomic analysis on *days 1*, *3*, *5*, and *7*–*10* of differentiation. *A*: number of detected and significantly regulated proteins for every timepoint comparing myotubes differentiated with (red) or without (blue) IGF1 at *days 1*–*10* (+IGF vs. −IGF) or the regulation vs. *day 1* in +IGF or −IGF. *B*: principal component analysis (PCA) for each sample in the proteomic analysis stratified by myotubes differentiated with (red) or without (blue) IGF1. *C*: PCA stratified by timepoint (colors) and myotubes differentiated without (light dot and color) and with (dark dot and color) IGF1. *D*: volcano plot depicting significantly different proteins between myotubes differentiated with or without IGF1 on *day 8* of differentiation. Colors additionally indicate significant regulation vs. *day 1* in +IGF (red = up; blue = down) and shapes indicate significant regulation vs. *day 1* in −IGF (triangle = up; square = down). The color black and shape circle indicate no regulation vs. *day 1*. Significant differences were defined by a Benjamini–Hochberg corrected *P* value below 0.05, *n* = 4 individual donors.

We used mass spectrometry-based proteomics to assess the time-dependent adaptations in the cellular proteome of the myotubes differentiated with or without IGF1. In total, 5,456 proteins were detected and only considered significantly changed at a corrected *P* value below 0.05 (BH) and when measured in at least three of four donors in one experimental group (Fig. 3*A*). During differentiation, the abundance of a comparable number of proteins was changed without IGF1 and in the presence of IGF1. Comparing +IGF versus −IGF samples at each timepoint, between 102 and 165 proteins showed higher abundance and between 88 and 139 showed lower abundance (Fig. 3*A*). Principle component analysis (PCA) over all samples revealed no outliers but a clear separation comparing +IGF versus −IGF along PC 2 (Fig. 3*B*). Color coded for group and day of differentiation, the PCA showed a clear separation along PC 1 with progressing differentiation from *day 1* to *day 10* as well as different trajectories for −IGF (*bottom left* to *top right*) and +IGF (*bottom left* to *bottom right*) (Fig. 3*C*). Interestingly, the proteome did not undergo further major changes within −IGF and +IGF groups between *day 7* and *day 10*. On *day 8*, both proteomes show the greatest distance from *day 1* indicating potentially fully differentiated myotubes. The proteome data suggest that the two different protocols lead to different proteomes showing that −IGF was incapable of differentiating toward the full phenotype of +IGF even if more time was given to fully differentiate (Fig. 3*C*). When looking at the top differentially abundant proteins comparing +IGF and −IGF on *day 8* we found many proteins important for skeletal muscle ATP supply (CKMT2, PYGM, PGAM2), overall muscular development (MLIP, CSRP3, MUSTN1, MYMK, FHL3), and contractile apparatus (MYL1, MYOM2, MYOM1, MYL2, MYBPC2, MYL11, TNNC2) (Fig. 3*D*). All these proteins were more abundant in myotubes differentiated +IGF.

### Elevated Skeletal Muscle-Associated Proteins in +IGF Myotubes

**Figure 4.**
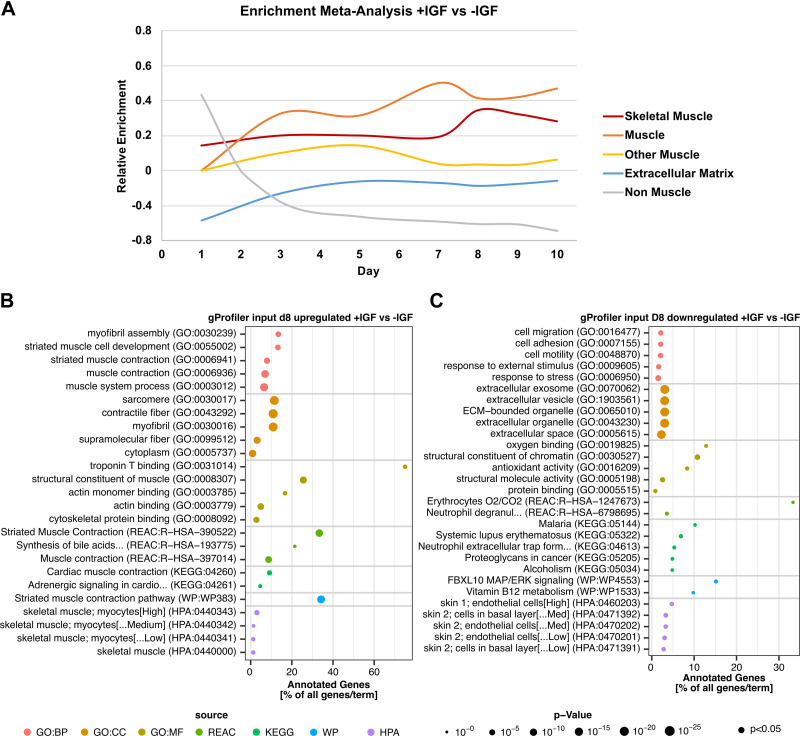
Pathway analysis during human myotube differentiation. Primary human myotubes differentiated in the presence or absence of insulin-like growth factor 1 (IGF1) were subjected to proteomic analysis over 10 days of differentiation. Pathway enrichment analysis was performed comparing myotubes +IGF vs. −IGF (*n* = 4 individual donors). *A*: enrichment meta-analysis, each top five enriched terms based on the up- or downregulated proteins comparing +IGF vs −IGF on each day was assigned to one of the preambles “Skeletal Muscle,” “Muscle,” “Other Muscle,” “Extracellular Matrix,” or “Non-Muscle” showing the quotient over the time of differentiation. *B*: gProfiler analysis based on significantly upregulated proteins on *day 8* +IGF vs −IGF. *C*: gProfiler analysis based on significantly downregulated proteins on *day 8* +IGF vs −IGF. Significant differences were defined by a Benjamini–Hochberg corrected *P* value below 0.05 and detection. GO:BP, gene ontology biological process; GO:CC, gene ontology cellular components; GO:MF, gene ontology molecular functions; HPA, human protein atlas; REAC, reactome; KEGG, Kyoto Encyclopedia of Genes and Genomes; WP, Wikipathways.

To get further insight into the proteomic changes induced by IGF1, we utilized enrichment analysis on the differentially abundant proteins comparing +IGF versus −IGF at each timepoint (Fig. 4). To generate an overview of the enrichment results for each day, we performed a meta-analysis. We assigned each top five enriched term based on the up- or downregulated proteins comparing +IGF versus −IGF on each day to one of the preambles “Skeletal Muscle,” “Muscle,” “Other Muscle,” “Extracellular Matrix,” or “Non-Muscle.” The quotient is displayed over the time of differentiation. Enriched terms associated with muscle in general, and specifically with skeletal muscle increased continuously over time in +IGF versus −IGF whereas other muscle associated terms decreased during the end of differentiation between *days 7* and *8*. Skeletal muscle-associated enriched proteins peak at *day 8* with IGF1. Terms associated with extracellular matrix (ECM) proteins were downregulated early during differentiation in +IGF and normalized at later timepoints (Fig. 4*A*).

On *day 8*, almost all enriched terms based on proteins with higher abundance in +IGF versus −IGF are associated with striated or skeletal muscle development, contractile apparatus, myofibril assembly, and striated muscle contractions (Fig. 4*B*) whereas the enriched terms based on proteins with lower abundance in +IGF versus −IGF are associated with non-muscle terms or the ECM (Fig. 4*C*). Well in accordance, activated upstream regulators comparing +IGF versus −IGF at *day 8* based on Ingenuity pathway analysis include muscle-specific transcription factors MEF2C, MYO1D, and ESSRA (Supplemental Fig. S2*A*). In summary, top regulated candidates and enrichment analysis comparing differentially abundant proteins in myotubes differentiated with or without IGF1 clearly hint toward more functional skeletal muscle differentiation and contractile apparatus assembly with IGF1. Based on the proteome data, *day 8* was chosen for further functional characterization.

### Elevated Contractile Apparatus Protein Expression in +IGF Myotubes

**Figure 5.**
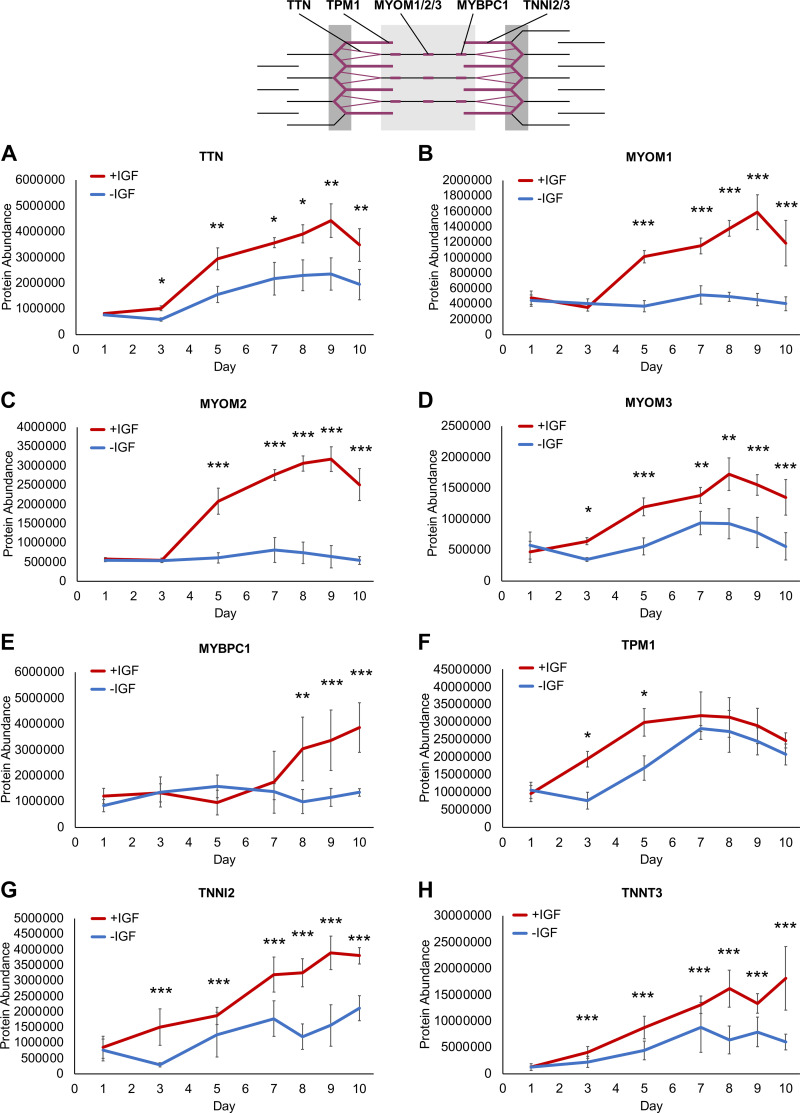
Contractile apparatus proteins during human myotube differentiation. Primary human myotubes differentiated in the presence or absence of insulin-like growth factor 1 (IGF1) were subjected to proteomic analysis over 10 days of differentiation. Schematic representation of the contractile apparatus generated with InkScape (v.1.0) highlights the location of analyzed proteins in purple. Regulation of contractile apparatus proteins titin (TTN; *A*), myomesin 1 (MYOM1; *B*), MYOM2 (*C*), MYOM3 (*D*), myosin binding protein C (MYBPC1; *E*), tropomyosin (TPM1; *F*), skeletal muscle troponin 2 (TNNI2; *G*), TNNT3 (*H*) was analyzed on *days 1*, *3*, *5*, *7*–*10* comparing protein levels between myotubes differentiated with or without IGF1. Curves represent means ± SD, based on median values over all detected peptides. Significant differences were defined by a Benjamini–Hochberg corrected *P* value below 0.05, *P < 0.05, **P < 0.01, ***P < 0.001, *n* = 4 individual donors.

As our proteome analysis clearly hinted toward striated muscle and contractile apparatus (Fig. 3*D*, and Fig. 4, *A* and *B*) and optical analysis showed proper contractile apparatus assembly with striated pattern after 8 days of differentiation with IGF1 (Fig. 2*A*), we looked into the protein expression of the contractile apparatus proteins in more detail (Fig. 5). Titin (TTN) connecting the myosins with the Z-band, the myomesins (MYOMs) connecting the myosins to form the M-band, myosin binding protein C (MYBPC1), tropomyosin (TPM1), and skeletal muscle troponins (TNNI2, TNNT3) that assemble the contractile apparatus were all elevated on protein level +IGF (Fig. 5, *A–H*). Although TTN, MYOM3, TPM1, TNNI2, and TNNT3 (Fig. 5, *A*, *D*, and *F*–*H*) were more upregulated +IGF, interestingly, MYOM1, MYOM2, and MYBPC1 (Fig. 5, *B*, *C*, and *E*) were only upregulated +IGF demonstrating insufficient supply of contractile apparatus proteins and thus potentially improper assembly of the contractile apparatus in the absence of IGF1.

### Similar Fast Type II Glycolytic Fiber Marker Proteins

**Figure 6.**
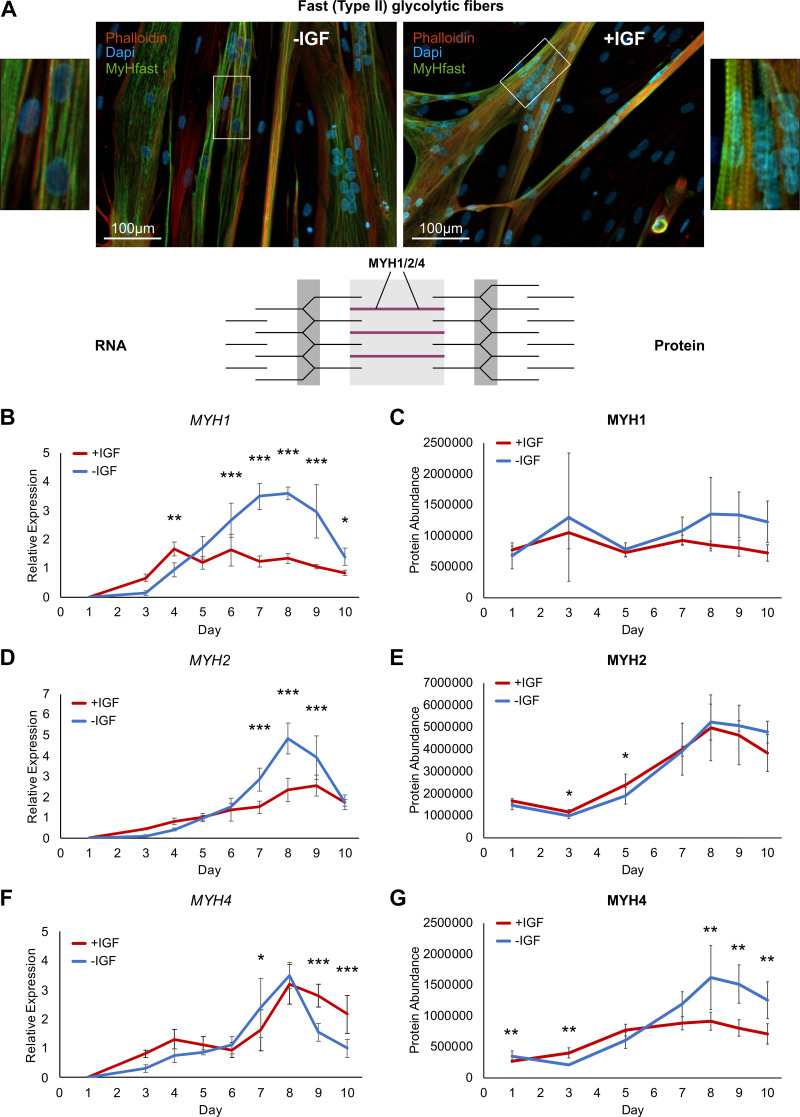
Fast (type II) glycolytic fiber markers during human myotube differentiation. Primary human myotubes differentiated in the presence or absence of insulin-like growth factor 1 (IGF1) were subjected to transcriptional and proteomic analysis over 10 days of differentiation. Schematic representation of the contractile apparatus generated with InkScape (v.1.0) highlights the location of analyzed proteins in purple. *A*: fixed slides of myotubes from a representative donor, differentiated with or without IGF1 on *day 8* of differentiation were stained against a myosin heavy chain fast (MyHfast, directed against MYH1 and MYH2) antibody (green), phalloidin (red) and DAPI (blue). Scale bars represent 100 µm. Regulation of fast (type II) glycolytic fiber markers *MYH1* (*B*), *MYH2* (*D*), *MYH4* (*F*) RNA expression was analyzed on *days 1*–*10* comparing expression between myotubes differentiated with or without IGF1. Curves represent means ± SD. Significant differences were assessed using one-way ANOVA with Fisher’s LSD post hoc test, *P < 0.05, **P < 0.01, ***P < 0.001, *n* = 4 individual donors. Regulation of fast (type II) glycolytic fiber marker proteins MYH1 (*C*), MYH2 (*E*), MYH4 (*G*) was analyzed on *days 1*, *3*, *5*, *7*–*10* comparing protein levels between myotubes differentiated with or without IGF1. Curves represent means ± SD, based on median values over all detected peptides. Significant differences were defined by a Benjamini–Hochberg corrected *P* value below 0.05, *P < 0.05, **P < 0.01, ***P < 0.001, *n* = 4 individual donors.

A key part of the contractile apparatus, also distinguishing the glycolytic from the oxidative fiber type in skeletal muscle, is the myosin heavy chains. We first looked at the fast glycolytic markers MYH1 and MYH2 and found intense staining in both myotubes from the same representative donor differentiated without and with IGF1 on *day 8* (Fig. 6*A*). Although sometimes a slight striated pattern was seen with the MyHfast antibody (directed against MYH1 and MYH2) in myotubes differentiated without IGF1, intracellular staining was still mostly diffuse. On the contrary, a clearly structured striated pattern was found with the MyHfast staining in myotubes differentiated with IGF1 (Fig. 6*A*). Next, we looked at the fast type II marker MYH1, MYH2, and MYH4 RNA and protein expression (Fig. 6, *B–G*). Although RNA expression of *MYH1* and *MYH2* showed lower levels in myotubes +IGF, protein abundance of MYH1 and MYH2 was rather comparable in +IGF versus −IGF (Fig. 6, *B–E*), similar to the immunohistochemistry staining (Fig. 6*A*). *MYH4* RNA levels were slightly elevated +IGF1 on *day 9* and *day 10* whereas MYH4 protein was less abundant in myotubes with IGF1 at the end of differentiation (Fig. 6*D*).

### More Slow Type I Oxidative Fiber Marker Proteins in +IGF Myotubes

**Figure 7.**
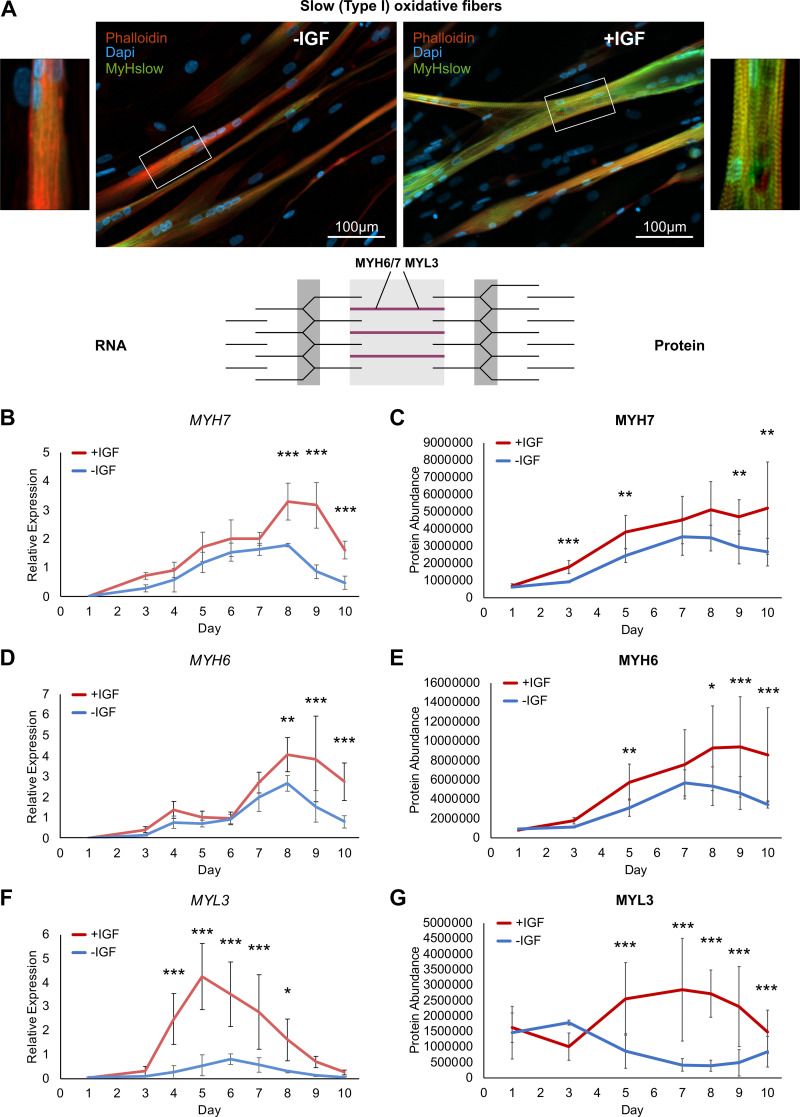
Slow (type I) oxidative fiber markers during human myotube differentiation. Primary human myotubes differentiated in the presence or absence of insulin-like growth factor 1 (IGF1) were subjected to transcriptional and proteomic analysis over 10 days of differentiation. Schematic representation of the contractile apparatus generated with InkScape (v.1.0) highlights the location of analyzed proteins in purple. *A*: fixed slides of myotubes from a representative donor, differentiated with or without IGF1 on *day 8* of differentiation were stained against a myosin heavy chain slow (MyHslow, directed against MYH7) antibody (green), phalloidin (red), and DAPI (blue). Scale bars represent 100 µm. Regulation of slow (type I) oxidative fiber markers *MYH7* (*B*), *MYH6* (*D*), *MYL3* (*F*) RNA expression was analyzed on *days 1*–*10* comparing expression between myotubes differentiated with or without IGF1. Curves represent means ± SD. Significant differences were assessed using one-way ANOVA with Fisher’s LSD post hoc test, *P < 0.05, **P < 0.01, ***P < 0.001, *n* = 4 individual donors. Regulation of slow (type I) oxidative fiber marker proteins MYH7 (*C*), MYH6 (*E*), MYL3 (*G*) was analyzed on *days 1*, *3*, *5*, *7*–*10* comparing protein levels between myotubes differentiated with or without IGF1. Curves represent means ± SD, based on median values over all detected peptides. Significant differences were defined by a Benjamini–Hochberg corrected *P* value below 0.05, *P < 0.05, **P < 0.01, ***P < 0.001, *n* = 4 individual donors.

Next, we studied the slow oxidative fiber type markers. In myotubes differentiated without IGF1, we found almost no myosin heavy chain slow (MyHslow) positive staining (directed against MYH7) on *day 8* indicating low abundance of MYH7 whereas myotubes differentiated with IGF1 from the same representative donor showed intense positive staining and again a clear striated pattern (Fig. 7*A*). In line with the immunohistochemical staining, RNA and protein expression of the most prominent oxidative type I marker MYH7 was stronger expressed throughout differentiation in myotubes differentiated with IGF1 (Fig. 7, *B* and *C*). Additional slow oxidative fiber type markers, MYH6 and MYL3 were also elevated on RNA and protein level especially later during differentiation (*day 5*–*10*) (Fig. 7, *D–G*). In summary, contractile apparatus proteins are more abundant in myotubes differentiated with IGF1 and histological assessment indicates proper assembly. Although fast glycolytic type II fiber type markers are present with both differentiation protocols, slow oxidative type II markers are only expressed or elevated in myotubes differentiated in the presence of IGF1.

### Elevated Energy Metabolism and Sarcoplasmic Ca^2+^ Release Proteins in +IGF Myotubes

**Figure 8.**
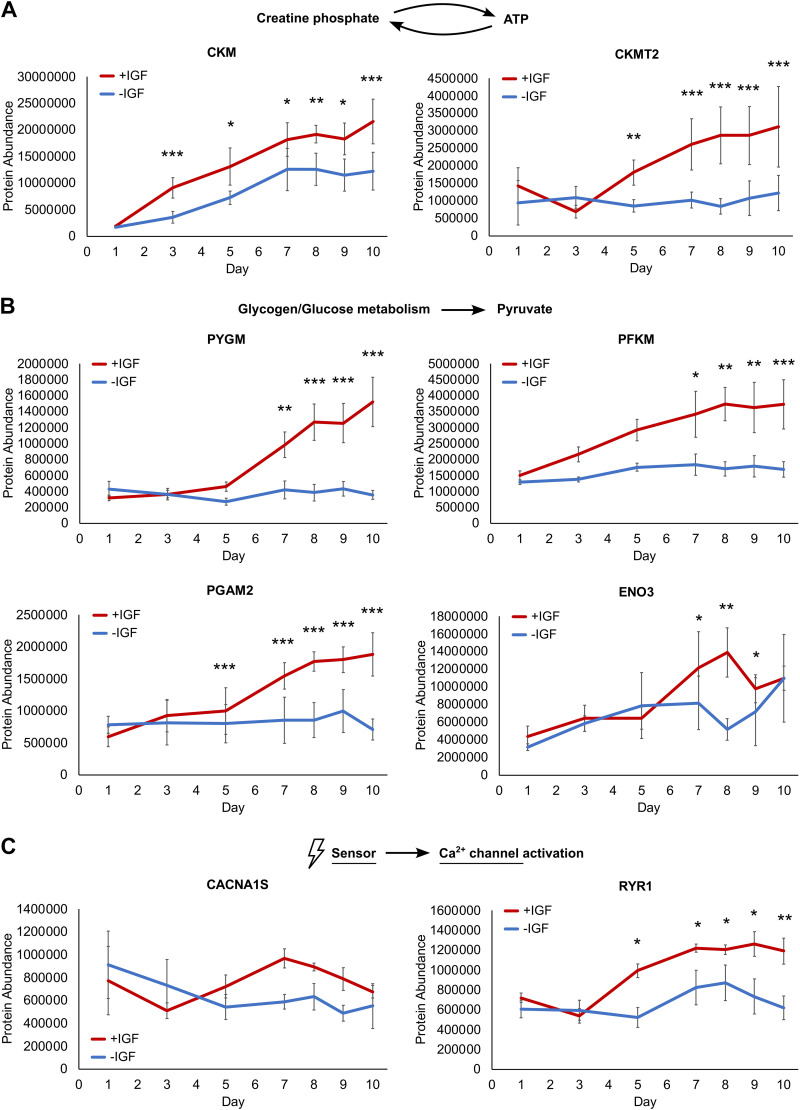
Energy metabolism and sarcoplasmic Ca^2+^ release proteins during human myotube differentiation. Primary human myotubes differentiated in the presence or absence of insulin-like growth factor 1 (IGF1) were subjected to proteomic analysis over 10 days of differentiation. Regulation of proteins relevant for ATP supply (CKM, CKMT2) (*A*), glycogenolysis and glycolysis (PYGM, PFKM, PGAM2, ENO3) (*B*), and voltage dependent Ca^2+^ release (CACNA1S, RYR1) (*C*) was analyzed on *days 1*, *3*, *5*, *7*–*10* comparing protein levels between myotubes differentiated with or without IGF1. Curves represent means ± SD, based on median values over all detected peptides. Significant differences were defined by a Benjamini–Hochberg corrected *P* value below 0.05, *P < 0.05, **P < 0.01, ***P < 0.001, *n* = 4 individual donors.

As we found enhanced contractility and expression of contractile apparatus proteins in +IGF myotubes, we next looked at proteins supporting energy supply during muscle contraction. Both striated muscle-specific creatine kinases, M-type (CKM) and mitochondrial 2 (CKMT2) catalyzing phosphate transfer from creatine phosphate to ATP were significantly more abundant starting at *days 3*–*5* in myotubes differentiated with IGF1 (Fig. 8*A*). Enzymes involved in glycogenolysis and glycolysis, namely glycogen phosphorylase (PYGM) and the rate-limiting glycolysis enzyme phosphofructokinase (PFKM) as well as downstream enzymes phosphoglycerate mutase 2 (PGAM2) and enolase 3 (ENO3) catalyzing conversion to pyruvate all were significantly elevated in +IGF myotubes (Fig. 8*B*). We also looked at the involved voltage sensor calcium voltage-gated channel subunit α1 s (CACNA1S) that triggers Ca^2+^ release from the sarcoplasmic reticulum via the calcium channel ryanodine receptor 1 (RYR1). Although elevated CACNA1S protein abundance from *day 5* in +IGF myotubes did not reach significance, RYR1 protein levels were significantly upregulated starting from *day 5* in myotubes differentiated in the presence of IGF1 (Fig. 8*C*). In summary, the abundance of proteins relevant for energy metabolism and sarcoplasmic Ca^2+^ release suggest that myotubes differentiated in the presence of IGF1 are better suited to cater to the demands of a physiological contraction.

### Elevated Mitochondrial Protein Expression and Mitochondrial Respiration in +IGF Myotubes

Since the new differentiation protocol utilizing IGF1 supports differentiation of myotubes presenting more oxidative type I markers, we investigated expression of all mitochondrial proteins comparing +IGF versus −IGF. Of the differentially expressed mitochondrial proteins between +IGF and −IGF at any stage of the differentiation (*day 1*–*10*), two-thirds were upregulated in myotubes differentiated with IGF1 (heatmap, *left*) and many of those were upregulated during differentiation compared with *day 1* (heatmap, *right*) (Supplemental Fig. S3*A*). Among the upregulated mitochondrial proteins are subunits of the respiratory chain complexes (Supplemental Fig. S3*B*).

**Figure 9.**
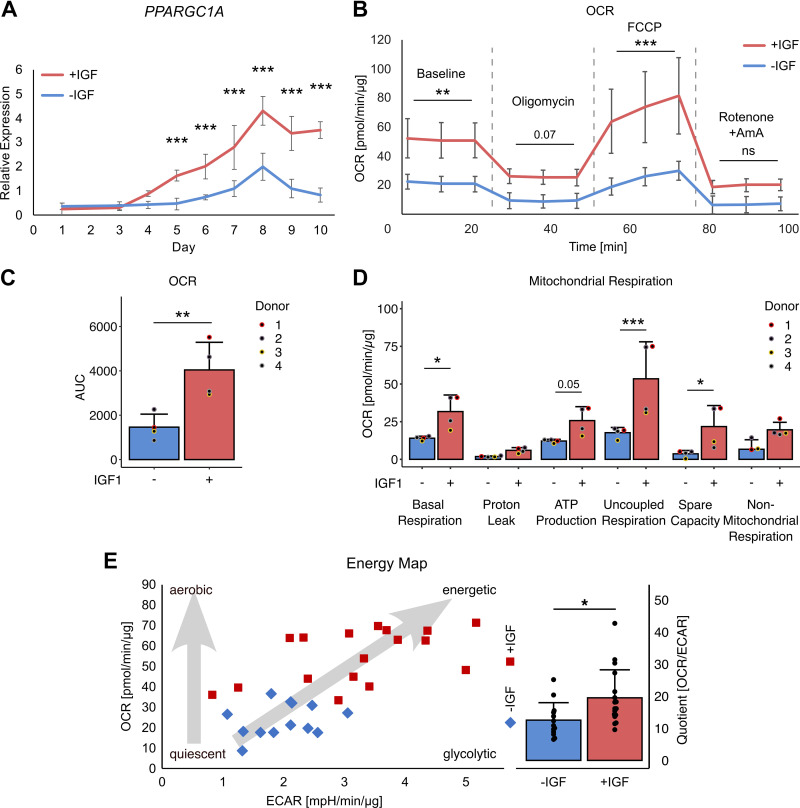
Mitochondrial respiration of human myotubes. Primary human myotubes were differentiated in the presence or absence of insulin-like growth factor 1 (IGF1) over 10 days. *A*: RNA expression of *PPARGC1A* was analyzed on *days 1*–*10* comparing expression between myotubes differentiated with or without IGF1. Curves represent means ± SD. *B*: respiration was measured in myotubes differentiated with and without IGF1 on *day 8* of differentiation in response to indicated substrates using seahorse analysis. Lines represent means ± SD of all analysis, labels describe oxygen consumption rate (OCR) measurements after single injections indicated by the dashed lines. For statistical analysis all measurements between injections were compared. Area under the curve was calculated for the complete measurements (*C*) and individual parameters of mitochondrial respiration calculated (*D*). *E*: using baseline measurement at the third timepoint, the energy map was drawn relating OCR and extracellular acidification rate (ECAR). The quotient was calculated and compared +IGF vs. −IGF. Significant differences were assessed using one-way ANOVA with Fisher’s LSD post hoc test, *P < 0.05, **P < 0.01, ***P < 0.001, *n* = 4 individual donors.

Both, fiber type markers and mitochondrial proteome signature hint toward differentiation of more oxidative fiber type myotubes. Well in line, expression of Peroxisome Proliferator-Activated Receptor Gamma Coactivator 1-Alpha (PGC1α/*PPARGC1A*) RNA, a key regulator of mitochondrial biogenesis, maintenance, and respiration, was strongly upregulated and elevated in myotubes differentiated with IGF1 starting from *day 5* with a peak at *day 8* (Fig. 9*A*). Thus, we next used seahorse analysis to measure oxygen consumption and mitochondrial respiration (Fig. 9, *B–D*). At all stages of the measurement, oxygen consumption rate (OCR) was elevated +IGF which was significant at baseline and with FCCP (Fig. 9*B*). The area under the curve over the complete measurement showed significantly elevated respiration in myotubes with IGF1 (Fig. 9*C*). Analyzing the respiratory states in detail, we found not only significantly elevated basal respiration but also more ATP production and uncoupled respiration as well as more spare capacity in myotubes differentiated in the presence of IGF1 (Fig. 9*D*). The energy map showed a shift from a quiescent to a metabolically active state in myotubes +IGF (Fig. 9*E*). The significantly elevated OCR/ECAR quotient indicates that this is more driven by the increase in mitochondrial respiration than in glycolysis (Fig. 9*E*). In summary, in line with more oxidative fiber type markers and mitochondrial protein expression, myotubes differentiated in the presence of IGF1 do not only contract in a functional manner but show elevated mitochondrial respiration.

### Insulin-Dependent Glucose Uptake in +IGF Myotubes

**Figure 10.**
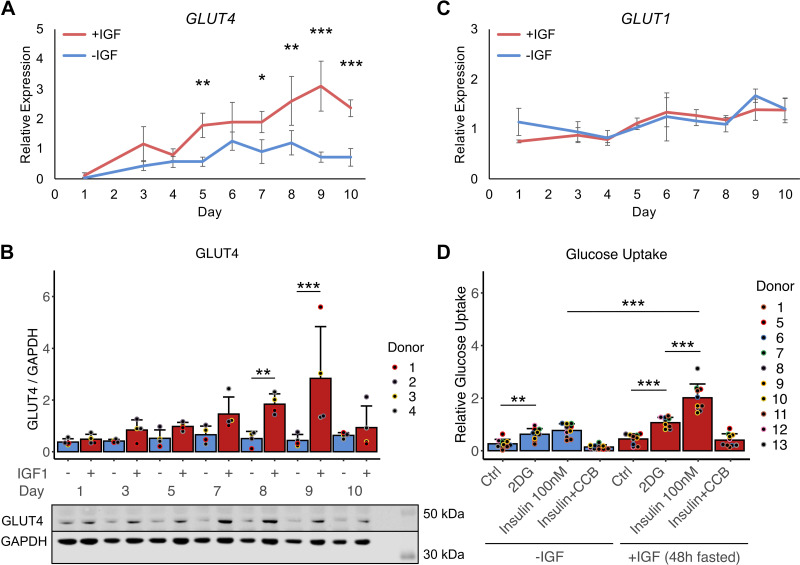
Glucose uptake of human myotubes. Primary human myotubes were differentiated in the presence or absence of insulin-like growth factor 1 (IGF1) over 10 days. RNA expression of *GLUT4* (*A*) and GLUT4 protein levels (*B*) by Western blotting were analyzed on *days 1*–*10* comparing myotubes differentiated with or without IGF1. *C*: *GLUT1* RNA expression was analyzed +IGF vs. −IGF over 10 days. Curves and bars represent means ± SD, *n* = 4 individual donors. *D*: glucose uptake, utilizing 2-deoxyglucose, was measured at baseline and in response to insulin stimulation in myotubes differentiated in the presence or absence of IGF1 on *day 8*. Bars represent means ± SD, individual data points are depicted, *n* = 10 individual donors. Significant differences were assessed using one-way ANOVA with Fisher’s LSD post hoc test, *P < 0.05, **P < 0.01, ***P < 0.001.

One important requirement for primary human myotubes as in vitro model for diabetes research is the proper expression of the insulin-dependent glucose transporter GLUT4. Therefore, we studied the impact of IGF1 on the relevant glucose transporters GLUT4 and GLUT1. Expression of *GLUT4* RNA was significantly elevated in myotubes with IGF1 starting at *day 5* of differentiation (Fig. 10*A*). On protein level, the same trend was observed with significantly elevated GLUT4 protein on *days 8* and *9* of differentiation in the presence of IGF1 (Fig. 10*B*). In contrast, GLUT4 RNA and protein expression stayed low in myotubes differentiated without IGF1 (Fig. 10*B*). Expression of GLUT1 RNA and protein was similar between myotubes +IGF and −IGF (Fig. 10*C*, Supplemental Fig. S4*A*). Glucose uptake using a 2-deoxyglucose assay was measured after withdrawal of IGF for 48 h, since the response to insulin shown as phosphorylation of AKT on serine-473 was impaired in myotubes that received IGF1 until they were stimulated with insulin (0 h, Supplemental Fig. S4, *B* and *C*). Fasting IGF1 for 48 h or 96 h recovered the insulin sensitivity of +IGF myotubes (48 h, 96 h, Supplemental Fig. S4, *B* and *C*). As expected from the comparable GLUT1 abundance (Fig. 10*A*, Supplemental Fig. S4), both +IGF and −IGF myotubes showed similar basal glucose uptake in the 2-deoxyglucose assay (Fig. 10*C*). Although myotubes differentiated without IGF1 were incapable of elevating glucose uptake after insulin stimulation, myotubes differentiated with the new protocol in the presence of IGF1 showed a twofold induction of glucose uptake after insulin stimulation. In summary, myotubes differentiated in the presence of IGF1 show functional contraction in line with elevated proper contractile apparatus protein assembly, differentiation of more oxidative fiber type myotubes in line with elevated mitochondrial respiration, and elevated GLUT4 protein in line with elevated insulin-dependent glucose uptake.

## DISCUSSION

In this study, we developed and characterized a culture system to achieve functionally active, contractile human myotubes suitable to study metabolic behavior and parameters relevant for exercise and diabetes research. Based on a serum-free system, the sole addition of IGF1 drives the cells toward more mature differentiated myotubes capable of contraction, insulin-dependent glucose uptake, and oxidative phosphorylation. We not only functionally validated the phenotype but demonstrated a pronounced shift in the proteome compared with myotubes differentiated without IGF1, which is responsible for the improved functionality.

Many growth factors are important for muscular development and myotube differentiation. Myotube growth, differentiation, and hypertrophy are primarily regulated by a signaling pathway initiated by insulin-like growth factor 1 (IGF1) (45). In vivo, IGF1 can be secreted by myofibers, macrophages, and endothelial cells in response to injury and exercise, or reach the muscles through the circulatory system inducing an intracellular cascade after binding its receptor (46, 47). IGF1 induces Akt signaling that via mTOR promotes protein synthesis and hypertrophy while inhibiting protein degradation and muscle atrophy by impeding GSK3 and the FOXO family of transcriptions factors (48, 49). The mammalian target of rapamycin (mTOR) signaling pathway also plays a key role in skeletal muscle differentiation by upregulating the expression of Pax7, Myf5, MyoD, and myogenin (50). Besides IGF1, HGF, FGF, and PDGF have been associated with muscle repair and hypertrophy by activation of satellite cells (51). However, all three, HGF, FGF, and PDGF had been shown to inhibit myogenic differentiation maintaining a proliferative phenotype of satellite cells and myoblasts (47, 52). Thus, for a growth factor to improve myotube differentiation we focused on IGF1 in this study.

IGF1 was previously investigated in human myotubes with regards to delaying or overcoming myoblast senescence and to induce hypertrophy with singular IGF1 doses or 1-h treatments (33, 53, 54). In these studies, IGF1 treatment was already described to increase myotube diameters and helped to overcome the detrimental effects of cotreatment with TNF-α on myotube differentiation (54). Reporting similar improvements in differentiation some studies also utilized insulin in their differentiation media (33, 54). Mostly for myotube differentiation from induced pluripotent stem cells (iPSC) or mesenchymal stromal cells (MSC), insulin containing ITS (Insulin-Transferrin-Selenium) is used with varying success (55, 56). Insulin is however known to hold the potential to render cells insulin resistant over culture periods (57, 58) making insulin or insulin-containing ITS supplementation unsuitable for a diabetes research model. Some studies focused on a specific isoform of IGF1 termed mechano-growth-factor or MGF (IGF1c in human, IGF1b in rodents) that is only detectable in tissue after mechanical strain and/or damage (59, 60). MGF was suggested to be very effective in promoting myotube differentiation in one study (61) only to be found less effective than recombinant IGF1 by another study reporting stronger improvement in myotube area and diameters with IGF1 than MGF (62). In this study, we further improved primary human myotube differentiation by supplementing human recombinant IGF1 in a serum- and insulin-free approach and extensively characterized the functional and metabolic phenotype of differentiated myotubes beyond the previously described benefits on diameter and hypertrophy.

Mimicking exercise in in vitro cultures utilizing EPS with primary human myotubes yields molecular responses that are in part relatable to in vivo responses of skeletal muscle to exercise (63, 64). However, often these studies fail to report a visually contracting phenotype or if provided, show diminutive degrees of movement likely due to the limitations of previous in vitro differentiation protocols and the use of serum (27, 28). In a previous study with the initial focus on coculture of human myotubes with motoneurons, Guo et al. (25) used a labor-intensive protocol with several distinct growth factor cocktails in subsequent use during primary human myotube culture. They focused on electrophysiological properties of the differentiated myotubes and reported spontaneous contractions in culture as well as EPS-induced contraction comparable with our myotubes. Their protocol contains two similarities to our approach. Apart from containing a mix of neurotrophins [brain-derived neurotrophic factor (BDNF), glial cell line-derived neurotrophic factor (GDNF), neurotrophin-3/4 (NT3/4), ciliary neurotrophic factor (CNTF)] and other additives, their differentiation media 2 and 3 contained no serum and a small amount of IGF1 (10 ng/mL). We also previously described the beneficial effects of using completely serum-free myotube differentiation with respect to the myosin heavy chain expression profile, PGC1α (*PPARGC1A*) expression levels, and number of nonfused cells already without IGF1 supplementation (24). By adding only IGF1 to our previous one-step serum-free differentiation protocol, we achieved a comparable degree of functional myotube differentiation already on *day 8* as previously reported for *day 10* (25). Furthermore, our proteomic analysis provides a comprehensive characterization and molecular basis for the capability to contract by revealing elevated levels of contractile apparatus proteins as well as proteins involved in energy metabolism such as ATP-supply, glycogenolysis, glycolysis, and voltage-dependent Ca^2+^ release from the sarcoplasm, essential for muscular contraction. In vitro, primary human myotubes are generally considered to have a lower mitochondrial oxidative capacity preferring glycolysis over lipid oxidation (26). Accordingly, human myotubes show elevated MYH1 and MYH2 expression over MYH7 that is characteristic for the glycolytic type II fiber type (26). We here found that IGF1-guided myotube differentiation led to upregulation of slow type I MYH7, MYH6, and MYL3 proteins suggesting that IGF1 drives myotube differentiation toward a more oxidative phenotype. Animal studies demonstrated the importance of PGC1α for fiber-type switching from glycolytic fast-type to oxidative slow-type muscle fibers (65, 66). Thus, the robust upregulation of PGC1α provides one potential explanation for the increased oxidative capacity of IGF1-treated myotubes, which was finally validated by analyzing mitochondrial respiration. Altogether this data shows that IGF1-guided differentiation of human myotubes shifts differentiation toward a more oxidative slow-type I fiber phenotype.

Another limitation of human myotubes in vitro was that, compared with in vivo, the insulin-stimulated glucose uptake appears impaired, accompanied by reduced GLUT4 expression at least in relation to GLUT1 (26). In vivo, GLUT4 is more highly expressed in type I oxidative muscle fibers that are generally regarded as having a greater insulin-stimulated glucose uptake (67, 68). Our IGF1-guided differentiation promoted development of more oxidative type I myotubes and in line, we found elevated GLUT4 mRNA and protein expression at similar GLUT1 levels. Because of the high similarity of insulin- and IGF1-receptors, the existence of hybrid receptors and the potential of IGF1 to bind to the insulin receptor although with lower affinity, combined with a substantial overlap of downstream signaling via IRS and AKT, it is possible that cells treated with IGF1 can become insulin resistant (69, 70). In line we saw reduced responsiveness to 10 nM insulin stimulation for 10 min in AKT phosphorylation in myotubes cultured with continuous IGF1 supplementation compared with 48 h or 96 h of IGF1 fasting. After 48 h of IGF1 fasting, myotubes differentiated with IGF1 were not only insulin responsive but also displayed a twofold elevated glucose uptake in response to insulin stimulation that myotubes differentiated without IGF1 were incapable of. Therefore, we were able to show that IGF1-guided myotube differentiation is able to improve the shortcomings of previous differentiation protocols regarding GLUT4 expression and glucose uptake.

We assume that the pronounced effect on functional myotube differentiation with our protocol can be attributed to the combination of a serum-free basal medium with IGF1 supplementation. The skeletal muscle- and contractile apparatus-specific protein expression is in line with the role of IGF1 in skeletal muscle development. The overall improvements compared with previous studies, sometimes also utilizing IGF1, are however likely observed due to a more unobstructed IGF1 activity in serum-free differentiation medium. Serum can contain other growth factors such as WNT, transforming growth factor (TGF)-β, or bone morphogenetic protein (BMP) agonists, that can induce antagonizing signaling pathways inhibiting the beneficial effects of IGF1 signaling, or IGF-binding proteins that directly bind and inhibit IGF1 (71, 72). Thus, our data show and give suggestions why IGF1-guided human myotube differentiation overcomes the previously inadequate capability of established human myotube models with our newly described simple one-step differentiation protocol.

We are aware that high levels of functionality can also be achieved and observed with 3-D culture tissue engineering approaches also giving way to even more elaborate readouts (73–75). However, it was our goal to develop an easy to use one-step two-dimensional (2-D) protocol that can be implemented in many laboratories, driven by metabolic or diabetes-related research questions, where monolayer myotube culture is readily available while establishing complicated tissue engineered 3-D solutions is no valid option. For these use cases our protocol allows studies with a more functional human myotube with the same culture methods most researchers are familiar with.

One limitation of this extensive characterization of an IGF1-guided human myotube differentiation is that the time course data due to handling limitations could only be performed in parallel in four donors that were all overweight. However, age and weight of the donors projected a substantial range and two males and two females were chosen. As qRT-PCR and proteomics data presented with robust results and low variation, and most functional analysis were repeated in additional donors we consider the data set robust and appropriate to underline our conclusions. Also, for functional analyses, like the insulin-dependent glucose uptake, donor number was increased to strengthen and validate our results. Nevertheless, future projects might be centered around characterizing differences in resting state and exercise response between cultured myotubes from healthy donors and donors with obesity, prediabetes, or diabetes using our new protocol. In addition, we only used one IGF1 concentration throughout the study. It is possible that the observed effects can be achieved with lower concentrations as previously also lower concentrations were reported to induce some effects on hypertrophy (33, 62). Thus, lower concentrations or also shorter periods of IGF1 treatment might be sufficient to induce the reported beneficial effects on differentiation.

### Conclusions

Utilizing IGF1 in a serum-free differentiation medium, we here describe that a simple, new one-step differentiation protocol generates functional primary human myotubes from isolated satellite cells. Compared with previous published protocols this approach can be easily adopted by anyone working with human myoblasts. The obtained myotubes within a week of differentiation recapitulate the physiological traits of skeletal muscle in vivo vastly superior to established 2-D in vitro protocols. Especially as a model for diabetes and exercise research, the capability to contract together with a more oxidative phenotype open up novel possibilities to study the molecular processes underlying the beneficial effects of exercise to prevent the onset of type 2 diabetes. Our results are also valuable for tissue engineering of linearly aligned myobundles in perfusable bioreactors and transfer to microfluidic systems for an exercise-on-chip model for drug screening and development of an interorgan cross talk model.

## DATA AVAILABILITY

The proteomic data used in this study are available via PRIDE—Proteomics Identification Database Project accession: PXD043200. Further data that support the findings of this study are available from the corresponding author upon request.

## SUPPLEMENTAL DATA

10.6084/m9.figshare.25288003 Supplemental Figs. S1–S4: https://doi.org/10.6084/m9.figshare.25288003.
Supplemental Videos S01–S14: https://doi.org/10.6084/m9.figshare.25288006.

## GRANTS

This study was supported in part by grants from the German Federal Ministry of Education and Research (BMBF) to the German Centre for Diabetes Research (DZD e.V.) under Grant No. 01GI0925. To fund this work, S.I.D. received the “Allgemeine Projektförderung der Deutschen Diabetes Gesellschaft (DDG) 2022.”

## DISCLOSURES

No conflicts of interest, financial or otherwise, are declared by the authors.

## AUTHOR CONTRIBUTIONS

S.I.D., P.G., and C.W. conceived and designed research; P.G., C.v.T., A.M., and J.M. performed experiments; S.I.D., C.v.T., T.G., and S.M.H. analyzed data; S.I.D., C.v.T., and C.W. interpreted results of experiments; S.I.D. prepared figures; S.I.D. and C.W. drafted manuscript; S.I.D. and C.W. edited and revised manuscript; S.I.D., P.G., C.v.T., A.M., J.M., T.G., A.L.B., A.P., P.L., S.M.H., and C.W. approved final version of manuscript.
